# Baseline Neutrophil–Lymphocyte and Platelet–Lymphocyte Ratios as Biomarkers of Survival in Cutaneous Melanoma: A Multicenter Cohort Study

**DOI:** 10.1245/s10434-018-6660-x

**Published:** 2018-07-31

**Authors:** Ryckie G. Wade, Alyss V. Robinson, Michelle C. I. Lo, Claire Keeble, Maria Marples, Donald J. Dewar, Marc D. S. Moncrieff, Howard Peach

**Affiliations:** 1 Faculty of Medicine and Health, Worsley Building, University of Leeds, Leeds, UK; 2 Department of Plastic and Reconstructive Surgery, Leeds General Infirmary, Leeds, UK; 3 Department of Plastic and Reconstructive Surgery, Norfolk and Norwich University Hospital NHS Trust, Norwich, UK; 4 Leeds Cancer Centre, St James’s University Hospital, Leeds, UK; 5 Norwich Medical School, University of East Anglia, Norwich, UK

## Abstract

**Background:**

In the peripheral blood, the neutrophil–lymphocyte ratio (NLR) and platelet–lymphocyte ratio (PLR) change in response to malignancy. These biomarkers are associated with adverse outcomes in numerous cancers, but the evidence is limited in relation to melanoma. This study sought to investigate the association between these biomarkers and survival in Stages I–III cutaneous melanoma.

**Methods:**

This multicenter cohort study investigated a consecutive series of patients who underwent wide excision of biopsy-proven cutaneous melanoma and sentinel lymph node biopsy during a 10-year period. The baseline NLR and PLR were calculated immediately before sentinel lymph node biopsy. Adjusted hazard ratios (HRs) for overall and melanoma-specific survival were generated.

**Results:**

Overall, 1351 patients were included in the study. During surveillance, 184 of these patients died (14%), with 141 of the deaths (77%) attributable to melanoma. Worse overall survival was associated with a baseline NLR lower than 2.5 [HR 2.2; 95% confidence interval (CI) 2.0 to 2.3; *p* < 0.001] and a baseline PLR lower than 100 (HR 1.8; 95% CI 1.7 to 1.8; *p* < 0.001). Melanoma-specific survival also was worse, with a baseline NLR lower than 2.5 (HR 1.9; 95% CI 1.6 to 2.2; *p* < 0.001) and a baseline PLR lower than 100 (HR 1.9; 95% CI 1.7 to 2.2; *p* < 0.001). The 5-year survival for patients with sentinel lymph node metastases and a low NLR and PLR was approximately 50%.

**Conclusion:**

This study provides important new data on biomarkers in early-stage melanoma, which contrast with biomarker profiles in advanced disease. These biomarkers may represent the host inflammatory response to melanoma and therefore could help select patients for adjuvant therapy and enhanced surveillance.

**Electronic supplementary material:**

The online version of this article (10.1245/s10434-018-6660-x) contains supplementary material, which is available to authorized users.

Cutaneous melanoma represents 7% of skin cancers but is responsible for 78% of skin cancer-related deaths. Its incidence in the Western world is rising faster than that of any other major cancer, and the United Kingdom has more than 15,000 new diagnoses annually.^1^

The survival rate is higher than 90% for Stage I and II disease, but the presence of regional or distant metastases reduces the 5-year relative survival rate to 50–55% for Stage I and to 8–25% for Stage II disease.^1^ Currently, the most powerful staging investigation for American Joint Committee on Cancer (AJCC) Stages IB to IIC melanoma (comprising approximately 55% of patients with invasive primaries)^2^ is sentinel lymph node biopsy (SLNB). Numerous algorithms to predict survival from clinical and histopathologic features exist, but these have modest diagnostic accuracy at best. It is well-established that the addition of host biomarkers to predictive models improves their accuracy, and an unmet need for these data in cutaneous melanoma remains.

The typical host response to malignancy involves neutrophilia, monocytosis, thrombophilia, and lymphocytopenia.^3^^–^6 Changes in these peripheral blood counts are best represented by their ratios, namely, the neutrophil–lymphocyte ratio (NLR), the platelet–lymphocyte ratio (PLR), and the lymphocyte-monocyte ratio (LMR). An increased NLR and PLR and a decreased LMR predict poor survival, recurrence, and response to therapy for many solid organ tumors.^7^^–^9 An abnormal baseline NLR is associated with adverse outcomes in advanced and high-risk melanoma,^10^^–^14 but has not been investigated for Stages I–III melanoma.

We hypothesized that NLR, PLR, and LMR baselines are associated with overall and melanoma-specific survival in Stages I–III cutaneous melanoma.

## Patients and Methods

### Study Design and Patients

This retrospective cohort study investigated consecutive patients who underwent wider re-excision (wide local excision [WLE]) of primary cutaneous melanoma and SLNB between 2006 and 2016 at the host institutions. A secure electronic database was prospectively completed by the direct clinical care team during the study period and retrospectively augmented using electronic hospital systems. Approval was gained from the research and ethical committees of Leeds Teaching Hospitals (reference PL15/368) and the National Health Research Authority for Norwich (IRAS project ID 234565).

Over 10 years, we included patients with a biopsy-proven primary cutaneous melanoma confirmed by study centre pathologists, who underwent WLE and SLNB. The following exclusion criteria ruled out patients with no full blood count (FBC) recorded between the time of initial biopsy and SLNB; the presence of factors that may have changed peripheral blood counts such as concurrent malignancy or infection, pregnancy, chronic inflammatory conditions, immunosuppressive medications, and proliferative hematopoietic disorders; and patients with multiple or occult primaries, second recurrences, or unidentifiable or unclassifiable tumors.

### Variables

All histopathologic features of the primary tumor (tumor diameter [mm], Breslow thickness [mm], mitotic count per mm^2^, the presence of ulceration, angiolymphatic and perineural invasion, microsatellites, tumor-infiltrating lymphocytes [TILs], and tumor regression) were recorded and changed accordingly where WLE yielded residual melanoma. Clinical stage was determined according to the seventh edition of the American Joint Committee on Cancer.^15^

We used the results of the baseline FBC (total white cell count; absolute neutrophil, lymphocyte, monocyte, and platelet counts) obtained after excision biopsy but before WLE and SLNB. If multiple blood tests were performed during this period, we used the result closest SLNB. Subsequently, NLR (absolute neutrophil ÷ absolute lymphocyte count), PLR (absolute platelet ÷ absolute lymphocyte count), and LMR (absolute lymphocyte ÷ absolute monocyte count) were computed.

### Outcomes

The primary outcome was survival. For overall survival, patients were censored if they were lost to follow-up or had died of any cause. For those who had died, melanoma-specific survival was defined as death directly attributable to melanoma.

### Sample Size

To achieve 90% power at 5% significance with a 4:1 sampling ratio (because 25% of cases have sentinel lymph node metastases^16^), we required 318 participants (*n*_1_ = 64, *n*_2_ = 254) to detect an overall survival hazard ratio of 1.5 using a two-tailed log-rank test.^10^^,^^17^^,^^18^ Because this was part of a larger study, we recruited more than the desired sample size to power other outcomes.

### Statistical Analysis

Optimal thresholds for NLR (2.5), PLR (100), and LMR (9) were determined via Cutoff Finder using the Manhattan distance.^19^ The risk for outcomes according to baseline biomarkers were estimated by complete case analyses using uni- and multivariable Cox regression to generate hazard ratios (HRs) and 95% confidence intervals (CIs) adjusted for clustering. Covariates for the multivariable models were known confounders and prescribed in our protocol. Log-rank statistics accompanied Kaplan–Meier plots. Models were internally validated with lossless non-parametric bootstrapping by resampling with replacement, with 1000 iterations, as per TRIPOD.^20^ All tests were two-sided, and significance was set at 5%.

## Results

**Fig. 1:**
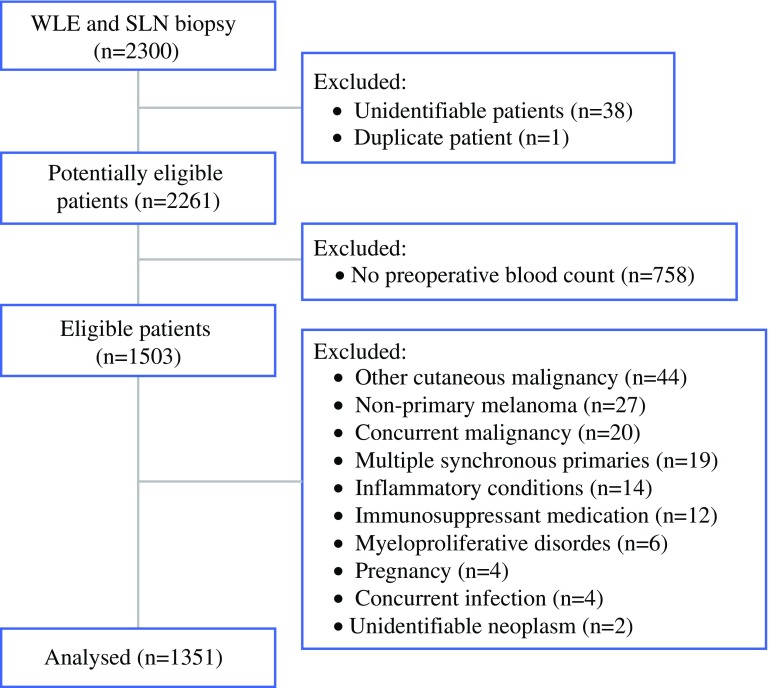
Participant flow diagram

For this study, 2300 patients were eligible, 1503 (65%) of whom had a relevant blood test. The median time from blood test to SLN biopsy was 4 days (interquartile range [IQR], 6 to 9 days). After 152 exclusions, 1351 patients (59%) remained for analysis (Fig. 1). The baseline characteristics are shown in Table 1. The geometric mean follow-up period was 3.5 years (95% CI 3.3 to 3.6 years; median, 3.8 years; minimum, 3 months; maximum, 10.7 years). During surveillance, 184 patients (14%) died, with 141 (77%) of those deaths attributable to melanoma.

**Table 1:** Baseline characteristics

	NLR < 2.5 (*n *= 789) *n* (%)	NLR ≥ 2.5 (*n *= 562) *n* (%)	*p* Value
Mean age (95% CI)^a^	59 (58 to 60)	63 (61 to 64)	<0.001
Sex (%)			
Male	368 (47)	310 (55)	0.002
Female	421 (53)	252 (45)	
Median Breslow thickness: mm (IQR)	1.9 (1.2 to 3.1; 0.3 to 24)	1.9 (1.2 to 3; 0.5 to 15)	0.9
Median mitoses: mm^−2^ (IQR)	3 (1 to 7)	3 (2 to 8)	0.6
Median maximum diameter: mm (IQR)	10 (7 to 14)	11 (7 to 15)	0.2
Ulceration	196 (27)	127 (24)	0.3
Angiolymphatic invasion	21 (6)	12 (5)	0.4
Perineural invasion	14 (4)	10 (4)	0.9
Regression	40 (12)	53 (21)	0.003
Microsatellites	15 (4)	14 (6)	0.6
Tumor-infiltrating lymphocytes			
Absent	53 (16)	39 (16)	0.2
Non-brisk	222 (69)	152 (63)	
Brisk	48 (15)	50 (21)	
Vertical growth phase	297 (97)	232 (99)	0.2
Pathologic subtype			
Nodular	87 (11)	57 (10)	N/A
Superficial spreading	230 (29)	179 (32)	
Acral	12 (2)	10 (2)	
Other	460 (58)	316 (56)	
Sentinel lymph node(s) containing metastatic melanoma	148 (19)	126 (22)	0.1
Extracapsular spread	12 (16)	4 (7)	0.08
AJCC Stage after sentinel lymph node biopsy			
I	518 (66)	372 (66)	0.04
II	121 (15)	63 (11)	
III	148 (18)	126 (22)	

*NLR* neutrophil–lymphocyte ratio; *CI* confidence interval; *IQR* interquartile ratio; *N/A* not applicable

^a^Geometric mean

The baseline blood counts of the survivors did not differ significantly from the counts of those who died, and no significant differences were observed in the absolute counts of neutrophils (median difference, 0.1; 95% CI − 0.3 to 0.2), lymphocytes (median difference, 0.1; 95% CI − 0.04 to 0.1), platelets (median difference, 6; 95% CI − 4 to 16), or monocytes (median difference, − 0.02; 95% CI − 0.04 to 0.1). This observation was similar for those who died of melanoma compared with survivors, as shown in Table 2.

**Table 2:** Overall and melanoma-specific survival for patients stratified according to peripheral blood ratios

Baseline biomarker			Crude risk		Adjusted risk^a^		
			HR (95% CI)	*p* value	HR (95% CI)	*p* value	Resampled^b^ *p* value
*Overall survival: individual biomarker*							
Continuous	NLR		1.1 (1.0 to 1.2)	0.07	0.8 (0.6 to 1.0)	0.03	< 0.001
	PLR		1.0 (1.0 to 1.0)	0.9	1.0 (1.0 to 1.0)	< 0.001	< 0.001
	LMR		1.0 (0.9 to 1.1)	0.6	1.0 (0.9 to 1.1)	0.8	0.6
Dichotomized	NLR	< 2.5	0.8 (0.6 to 1.1)	0.2	2.2 (2.0 to 2.3)	< 0.001	< 0.001
		≥ 2.5	1 (referent)		1 (referent)		
	PLR	< 100	1.2 (0.9 to 1.8)	0.2	1.8 (1.7 to 1.8)	< 0.001	< 0.001
		≥ 100	1 (referent)		1 (referent)		
	LMR	< 9	1 (referent)	0.3	1 (referent)	0.4	0.03
		≥ 9	1.5 (0.6 to 3.8)		1.3 (0.6 to 2.6)		
*Overall survival: compound biomarker*							
	NLR-low and PLR-low		1.1 (0.8 to 1.6)	0.02	3.0 (2.4 to 3.7)	< 0.001	< 0.001
	NLR-high or PLR-high		0.7 (0.5 to 0.9)		1.7 (1.1 to 2.7)		
	NLR-high and PLR-high		1 (referent)		1 (referent)		
*Melanoma-specific survival: individual biomarker*							
Continuous	NLR		1.1 (0.9 to 1.2)	0.7	0.8 (0.6 to 1.0)	0.07	< 0.001
	PLR		1.0 (1.0 to 1.0)	0.6	1.0 (1.0 to 1.0)	0.05	< 0.001
	LMR		1.0 (0.9 to 1.1)	0.9	0.9 (0.9 to 1.0)	0.5	0.2
Dichotomized	NLR	< 2.5	0.9 (0.7 to 1.3)	0.8	1.9 (1.6 to 2.2)	< 0.001	< 0.001
		≥ 2.5	1 (referent)		1 (referent)		
	PLR	< 100	1.4 (0.9 to 2.0)	0.1	1.9 (1.7 to 2.2)	< 0.001	< 0.001
		≥ 100	1 (referent)		1 (referent)		
	LMR	< 9	1 (referent)	0.8	1 (referent)	0.9	0.9
		≥ 9	1.2 (0.4 to 3.8)		0.9 (0.2 to 4.8)		
*Melanoma-specific survival: compound biomarker*							
	NLR-low and PLR-low		1.3 (0.9 to 2.0)	0.045	3.0 (2.0 to 4.2)	< 0.001	< 0.001
	NLR-high or PLR-high		0.8 (0.5 to 1.1)		1.4 (0.8 to 2.5)		
	NLR-high and PLR-high		1 (referent)		1 (referent)		

*HR* hazard ratio; *CI* confidence interval; *NLR* neutrophil–lymphocyte ratio; *PLR* platelet–lymphocyte ratio; *LMR* lymphocyte-monocyte ratio

^a^Each biomarker was examined individually by Cox regression, with adjustment, for age, Breslow thickness (mm), and mitotic rate (per mm^2^) as continuous variables; and for sex, ulceration, vascular invasion, tumor-infiltrating lymphocytes (TILs), regression, microsatellites, and sentinel lymph node involvement as categorical variables. CIs are adjusted for clustering

^b^Lossless non-parametric bootstrapping by resampling with replacement, with 1000 iterations. Low NLR was defined as < 2.5, whereas high NLR was defined as ≥ 2.5. Low PLR was defined as < 100, whereas high PLR was defined as ≥ 100

We observed no univariable associations between biomarkers and survival. After adjustment for confounders, multivariable analyses showed that a baseline NLR lower than 2.5 was associated with worse overall survival (adjusted HR 2.2; 95% CI 2.0 to 2.3) and worse melanoma-specific survival (adjusted HR 1.9; 95% CI 1.6 to 2.2; Table 2), meaning that patients with a low baseline NLR were at twice the risk of death during a 10-year period. A similar association remained when the biomarker was modeled as a continuous predictor, whereby for every unit increase in NLR, the risk of death decreased by 20%.

Similarly, an adjusted baseline PLR lower than 100 was significantly associated with worse overall survival (HR 1.8; 95% CI 1.7 to 1.8) and melanoma-specific survival (HR 1.9; 95% CI 1.7 to 2.2; Table 2), meaning that patients with a low baseline PLR were at approximately twice the risk of death from melanoma during a 10-year period. In addition, PLR was significantly associated with the outcome when modeled as a continuous predictor, although the increments were too small to be clinically meaningful.

**Fig. 2:**
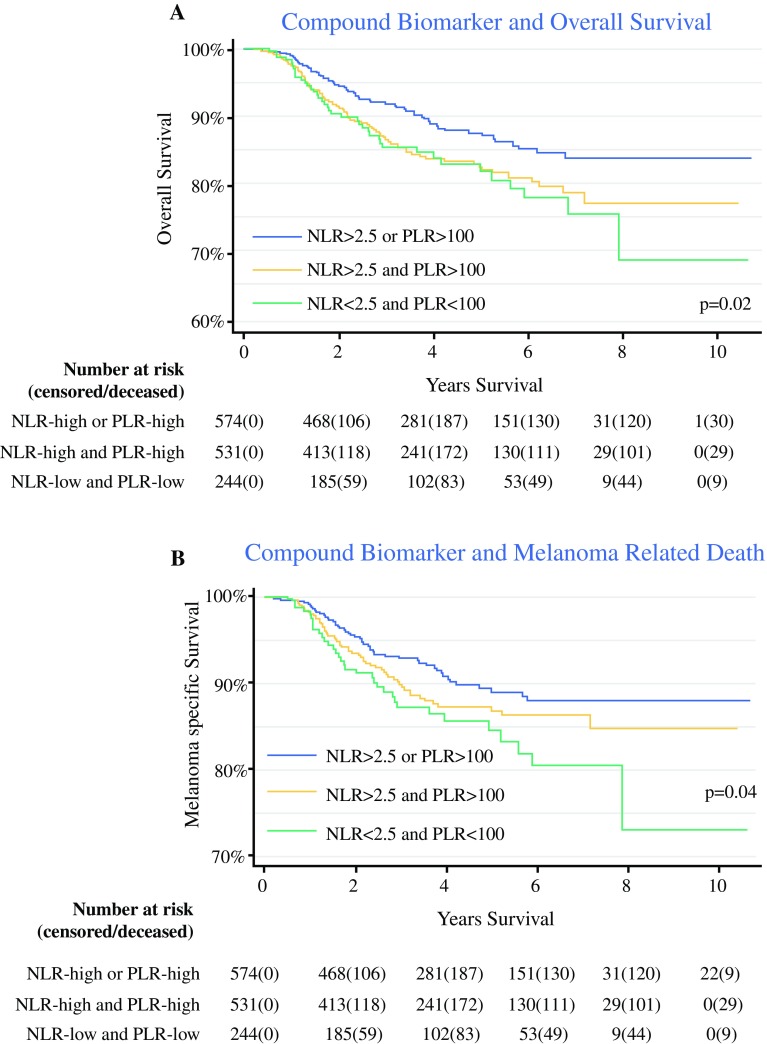
Kaplan-Meier plots of **a** overall and **b** melanoma-specific survival according to the compound biomarker of the neutrophil–lymphocyte ratio (NLR) and the platelet–lymphocyte ratio (PLR)

The patients with a low NLR and PLR were at three times the risk of death during a 10-year period (HR 3.0; 95% CI 2.4 to 3.7; Fig. 2a), overall and from melanoma specifically (HR 3.0; 95% CI 2.0 to 4.2; Fig. 2b). This suggests that patients with both a low NLR and a low PLR are at the greatest risk of death. All associations were strengthened by bootstrapping (Table 2). The associations between known clinicopathologic factors and overall survival are available online in the supplementary material (Table 3).

**Fig. 3:**
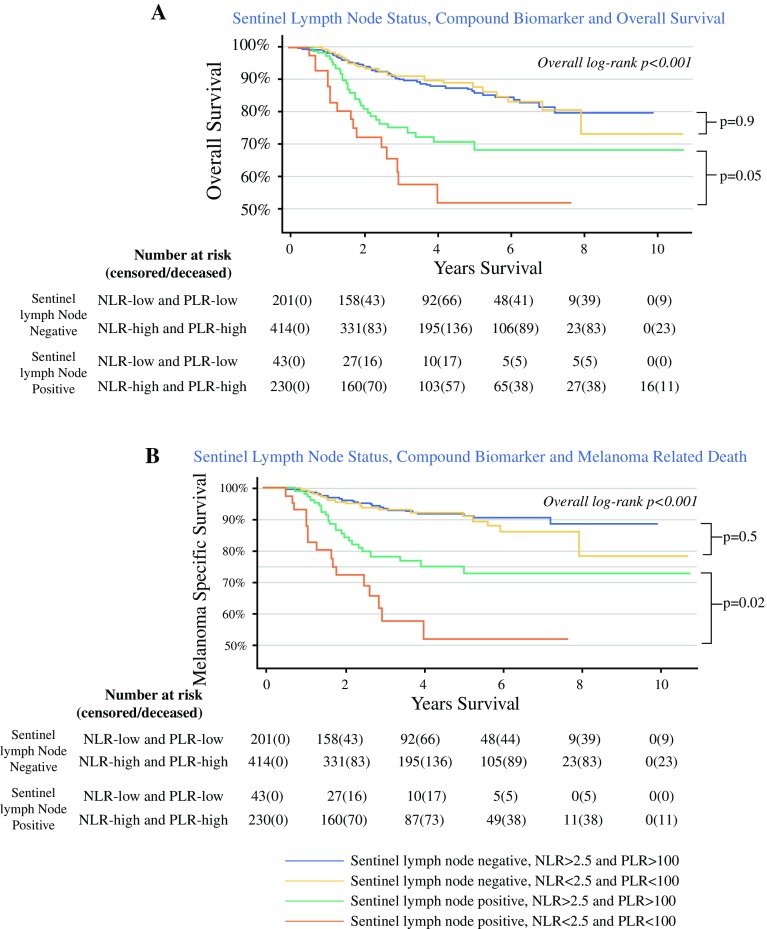
Kaplan-Meier plots of **a** overall and **b** melanoma-specific survival according to sentinel lymph node status and baseline compound biomarker

Stratification of the cohort by SLNB status showed that those with nodal disease and a low NLR and PLR have significantly worse overall survival (Fig. 3a) and melanoma-specific survival (Fig. 3b). The presence of sentinel lymph node metastases alongside a low baseline NLR and PLR was associated with four times the risk of death (HR 4.4; 95% CI 2.3 to 8.3; *p* < 0.001; Fig. 3a, orange line), and the median overall survival was 8 months less (IQR 2 to 15 months) than for the patients with a high NLR and PLR (Fig. 3a, green line). Similarly, nodal metastases with a low NLR and PLR were associated with almost six times the risk of death from melanoma (HR 5.8; 95% CI 3.0 to 11.5; *p* < 0.001; Fig. 3b, orange line), and the median melanoma-specific survival was 7 months less (IQR 2 to 15 months) than for the patients with a high NLR and PLR (Fig. 3b, green line).

The patients with regression of their primary tumor had a significantly higher median NLR (median difference, 0.3; IQR 0.1 to 0.5; *p* = 0.02) and a lower median LMR (median difference, 0.4; IQR 0.1 to 0.8; *p* = 0.02). However, no significant associations were observed between the NLR, PLR or LMR and the TILs status of the primary tumor.

## Discussion

This retrospective multicenter cohort study provided evidence of the potential prognostic and clinical utility of baseline hematologic biomarkers in cutaneous melanoma despite the lack of data for patients’ performance status and comorbidities. We showed that low neutrophil–lymphocyte and platelet–lymphocyte ratios, taken at the time of definitive treatment for the primary tumor, were associated with more than twice the risk of death from melanoma. Moreover, the findings showed that SLNB-positive patients can be stratified according to their NLR and PLR, which could potentially help clinicians identify patients who may benefit from adjuvant systemic therapy and enhanced surveillance. Our findings concur with the evolving hypothesis that host immunity is implicated in the survival of patients with melanoma, whereby a pro-inflammatory state may suppress or eradicate metastasis, explaining our observed better survival of those with a high NLR (i.e., a host inflammatory response).

Related research on melanoma survival and these biomarkers has considered only advanced disease, whereas we present data that applies to a greater proportion of patients with early-stage melanoma. Davis et al.^14^ showed that a baseline NLR higher than 3 was associated with a 25% increased risk of all-cause mortality. However, their study considered patients with pT2b or worse tumors and those presenting with nodal metastases. Accordingly, the proportion of their cohort with Stage III disease was substantially greater than ours (49 vs 18%). Although Lino-Silva et al.^13^ showed that an elevated baseline NLR (≥ 2) was associated with 31% worse survival, lymph node metastasis, and a higher risk of recurrence (although the statistics for recurrence were not provided), 96% of their cohort had acral lentiginous melanoma, and 41% had Stage III disease.

Finally, several studies of immunotherapies for Stage IV melanoma have shown an elevated baseline NLR to be associated with adverse outcomes.^10^^–^12 Unlike Davis et al.^14^ and Lino-Silva et al.,^13^ we found no univariable association between NLR and survival. However, after adjusting for confounding, we observed strong associations between NLR/PLR and survival, but in the opposite direction of the effect in the literature. This could mean that elimination of confounding is important in early disease. Nonetheless, our data add to the evolving literature on biomarkers in melanoma by providing important and new data on patients with earlier-stage disease, which contradicts the findings for Stage III^13^^,^^14^ and Stage IV melanoma.^10^^–^12^,^^14^^,^^18^ We hypothesise that these biomarkers may have a different prognostic role for patients with localized disease, whereby a high NLR may be favorable by indicating an inflammatory response to the melanoma.

Our estimates may be biased by selection because we do not know why some patients had preoperative blood tests, whereas others did not. Many individuals described in this cohort study also were enrolled in an observational study of the “normal” melanoma population^23^ that required FBC monitoring. Therefore, uncertainty notwithstanding, we believe this improves the external validity of our findings. Equally, we did not measure factors associated with melanoma outcome and biomarkers (e.g., obesity or ethnicity), which may have confounded our estimates, explaining why our findings contrast with the literature. Overall, the literature is deficient in peripheral blood biomarker research concerning localized skin cancer, and we believe the wealth of data on biomarkers in advanced cancers should not be generalised to early melanoma without further research.

The role of systemic inflammation in tumor progression has been widely researched. Despite a wealth of literature indicating a prognostic role of hematologic markers in cancer, including two large systematic reviews of more than 100 studies,^7^^,^^21^ few have sought to establish the underlying inflammatory mechanism for these changes. In healthy individuals, NLR but not PLR is associated with systemic C-reactive protein (CRP) and interleukin 6 (IL-6) levels,^22^ but the concordance between cytokines and peripheral blood counts in outcome prediction remains to be investigated. Furthermore, these processes may be unique to tumor type, and currently, no published works exist that implicate specific cytokines in melanoma. Widespread systemic inflammation in response to tumor burden in metastatic disease could explain the associations found between NLR and adverse outcomes for patients with disseminated melanoma,^10^^–^12^,^^18^ but the reversed association in our study suggests that inflammatory changes present in localized disease may be protective and, more importantly, detectable in the peripheral blood. Whether this subtle systemic inflammatory response (raised NLR and PLR) protects against melanoma metastasis or occurs in response to identified dissemination remains to be determined.

We have shown that decreased peripheral lymphocyte counts (as the NLR and LMR) are associated with regression of the primary melanoma, which is difficult to explain, particularly in the absence of any associations with peripheral lymphocyte counts or the presence of TILs in the primary tumor. This observation requires further research.

## Conclusion

The baseline NLR and PLR are significantly associated with disease-specific and overall survival in cutaneous melanoma. These biomarkers could potentially be useful in routine clinical practice and future clinical trials to identify patients who may benefit from adjuvant therapy and enhanced surveillance.

## Electronic supplementary material

Below is the link to the electronic supplementary material.

Supplementary material 1 (DOCX 20 kb)
