# Supplementary material for: Baseline Neutrophil–Lymphocyte and Platelet–Lymphocyte Ratios as Biomarkers of Survival in Cutaneous Melanoma: A Multicenter Cohort Study
Source: Ann Surg Oncol. 2018 Jul 31;25(11):3341–9. doi: 10.1245/s10434-018-6660-x (PMC6132419; doi:10.1245/s10434-018-6660-x)
Supplement: Supplementary file 1 — Supplementary material 1 (DOCX 20 kb) [file 10434_2018_6660_MOESM1_ESM.docx]

**Supplementary material**

| **Table 3. Overall survival** | | **Crude Risk** | | **Adjusted* Risk** | | |
| --- | --- | --- | --- | --- | --- | --- |
|  |  | **HR (95% CI)** | ***p*-value** | **HR (95% CI)** | ***p*-value** | ***Resampled^§^ p-value*** |
| Age in years | | 1.0 (1.0, 1.0) | <0.001 | 1.0 (1.0, 1.0) | 0.9 | 0.6 |
| Male sex | | 1.3 (1.0, 1.8) | 0.06 | 1.0 (1.0, 1.1) | <0.001 | <0.001 |
| Breslow thickness in mm | | 1.1 (1.1, 1.2) | <0.001 | 1.1 (1.0, 1.2) | 0.3 | 0.4 |
| Mitoses per mm^2^ | | 1.0 (1.0, 1.0) | <0.001 | 1.0 (1.0, 1.1) | 0.6 | 0.1 |
| Tumour diameter in mm | | 1.0 (1.0, 1.1) | 0.03 | / | / | / |
| Ulceration | | 2.3 (1.7, 3.2) | <0.001 | 2.2 (0.8, 6.1) | 0.2 | <0.001 |
| Angiolymphatic invasion | | 3.3 (1.8, 6.0) | <0.001 | 1.7 (1.0, 2.6) | 0.03 | <0.001 |
| Perineural invasion | | 0.8 (0.2, 2.7) | 0.6 | / | / | / |
| Regression | | 0.9 (0.5, 1.6) | 0.7 | 1.2 (0.6, 2.4) | 0.7 | 0.4 |
| Microsatellites | | 1.3 (0.5, 3.1) | 0.6 | 0.4 (0.2, 0.7) | 0.7 | <0.001 |
| Tumour infiltrating lymphocytes | Absent | 3.5 (1.3, 9.7) | 0.02 | 2.8 (2.3, 3.4) | 0.009 | <0.001 |
|  | Non-brisk | 3.6 (1.4, 9.0) |  | 2.9 (1.3, 6.3) |  |  |
|  | Brisk | 1 (referent) |  | 1 (referent) |  |  |
| Vertical growth phase | | 1.7 (1.3, 2.6) | <0.001 | / | / | / |
| Pathological subtype | Superficial spreading | 1 (referent) | 0.001 | / | / | / |
|  | Nodular | 2.0 (1.3, 3.1) |  | / |  |  |
|  | Other | 1.4 (0.8, 2.6) |  | / |  |  |
| Sentinel  lymph nodes containing  metastatic melanoma | 0 | 1 (referent) | <0.001 | 1 (referent) | 0.004 | <0.001 |
|  | 1 | 2.9 (1.5, 5.5) |  | 1.9 (1.2, 3.0) |  |  |
|  | ≥2 | 3.9 (1.8, 8.3) |  | 3.2 (3.1, 3.2) |  |  |

*Cox regression of overall survival adjusting for age, Breslow thickness (mm) and mitotic rate (per mm^2^) as continuous variables; sex, ulceration, vascular invasion, TILs, regression, microsatellites and sentinel lymph node involvement as categorical variables. CIs are adjusted for clustering.

***^§^***Lossless non-parametric bootstrapping by resampling with replacement, with 1000 iterations.
